# *In silico* screening for candidate chassis strains of free fatty acid-producing cyanobacteria

**DOI:** 10.1186/s12864-016-3389-4

**Published:** 2017-01-05

**Authors:** Olaa Motwalli, Magbubah Essack, Boris R. Jankovic, Boyang Ji, Xinyao Liu, Hifzur Rahman Ansari, Robert Hoehndorf, Xin Gao, Stefan T. Arold, Katsuhiko Mineta, John A. C. Archer, Takashi Gojobori, Ivan Mijakovic, Vladimir B. Bajic

**Affiliations:** 1 Computational Bioscience Research Center (CBRC), King Abdullah University of Science and Technology (KAUST), Thuwal, 23955-6900 Kingdom of Saudi Arabia; 2 Division of Systems & Synthetic Biology, Department of Biology and Biological Engineering, Chalmers University of Technology, Kemivägen 10, 41296 Gothenburg, Sweden; 3 SABIC Corporate Research and Development (CRD), Thuwal, 23955-6900 Kingdom of Saudi Arabia; 4 Pathogen Genomics Laboratory, Biological and Environmental Sciences and Engineering (BESE) Division, King Abdullah University of Science and Technology (KAUST), Thuwal, 23955-6900 Kingdom of Saudi Arabia

**Keywords:** Cyanobacteria, Free fatty acids, Biofuel, Screening method, Cell factories, Bioinformatics, Optimization, Computer science

## Abstract

**Background:**

Finding a source from which high-energy-density biofuels can be derived at an industrial scale has become an urgent challenge for renewable energy production. Some microorganisms can produce free fatty acids (FFA) as precursors towards such high-energy-density biofuels. In particular, photosynthetic cyanobacteria are capable of directly converting carbon dioxide into FFA. However, current engineered strains need several rounds of engineering to reach the level of production of FFA to be commercially viable; thus new chassis strains that require less engineering are needed. Although more than 120 cyanobacterial genomes are sequenced, the natural potential of these strains for FFA production and excretion has not been systematically estimated.

**Results:**

Here we present the **FFA SC** (FFASC), an *in silico* screening method that evaluates the potential for FFA production and excretion of cyanobacterial strains based on their proteomes. A literature search allowed for the compilation of 64 proteins, most of which influence FFA production and a few of which affect FFA excretion. The proteins are classified into 49 orthologous groups (OGs) that helped create rules used in the scoring/ranking of algorithms developed to estimate the potential for FFA production and excretion of an organism. Among 125 cyanobacterial strains, FFASC identified 20 candidate chassis strains that rank in their FFA producing and excreting potential above the specifically engineered reference strain, *Synechococcus* sp. PCC 7002. We further show that the top ranked cyanobacterial strains are unicellular and primarily include *Prochlorococcus* (order *Prochlorales*) and marine *Synechococcus* (order *Chroococcales*) that cluster phylogenetically. Moreover, two principal categories of enzymes were shown to influence FFA production the most: those ensuring precursor availability for the biosynthesis of lipids, and those involved in handling the oxidative stress associated to FFA synthesis.

**Conclusion:**

To our knowledge FFASC is the first *in silico* method to screen cyanobacteria proteomes for their potential to produce and excrete FFA, as well as the first attempt to parameterize the criteria derived from genetic characteristics that are favorable/non-favorable for this purpose. Thus, FFASC helps focus experimental evaluation only on the most promising cyanobacteria.

**Electronic supplementary material:**

The online version of this article (doi:10.1186/s12864-016-3389-4) contains supplementary material, which is available to authorized users.

## Background

The grand challenges of the 21^st^ century include fulfilling increasing demands for food, feedstock and chemical raw materials. As potential feedstock for renewable energy, the use of microbes that produce free fatty acid (FFA) has been strongly suggested [1–5]. Substantial efforts have been made to engineer *Escherichia coli* (*E. coli*) for FFA production [6–9]. However, when *E. coli* produces FFA, it requires fixed carbon sources that are too costly to be exploited as feedstock. As an alternative, lignocellulosic biomass was also considered as a feedstock, however this process demands huge amounts of fresh water and farmland [10, 11]. Thus, photosynthetic cyanobacteria and microalgae that directly convert carbon dioxide into FFA are seen as more promising alternatives. In comparison to microalgae, cyanobacteria can be more easily genetically engineered because they have smaller and less complex genomes, and are often naturally competent for DNA uptake [11]. Moreover, cyanobacteria have the ability to excrete FFA that simplifies the biomass extraction process thereby reducing total cost by at least 70% [12].

There are several aspects to consider when evaluating the potential of a cyanobacterial strain as a candidate chassis strain for FFA production in the context of biofuel production. Some of these aspects include: 1/native biosynthetic capability for FFA production and excretion, 2/environmental robustness, 3/strain turnover rate, 4/the necessary gene expression levels, 5/metabolic fluxes, and 6/established genetic engineering tools. The primary aspect to consider is the strain’s natural potential to produce and excrete FFA, as when this potential is weak the strain would be considered as less useful. For simplicity in what follows we will refer to ‘FFA production and excretion’ as ‘FFA production’. In cyanobacteria, fatty acids are synthesized via the type II fatty acid synthases (FAS). Focal to fatty acids synthesis are acyl carrier protein (ACP) that covalently binds all fatty acyl intermediates during the synthesis process. Fatty acid synthesis represents a central, conserved process by which acyl chains are produced and core enzymes required for fatty acids initiation and elongation are well characterized [12, 13]. FFA production has been investigated in several cyanobacterial strains including *Synechococcus* sp. PCC 7002 [14], *Synechocystis* PCC 6803 [12, 15, 16], *Synechococcus elongatus* PCC 7942 [17] and *Arthrospira* (*Spirullina) platensis* NISE-39 [18, 19]. Of these cyanobacterial strains, the model system *Synechocystis* PCC 6803 has received the most research attention because of its ability to grow photoautrophically and heterotrophically. Moreover, it was the first cyanobacterial genome to be completely sequenced [20, 21]. Current applications of cyanobacteria for sustainable production focus on utilizing different metabolic engineering strategies to maximize FFA production [22]. However, current engineered strains are not producing sufficient amounts of FFA to be commercially viable. To optimize overproduction of desired products such as fatty acids (*E. coli*) [23], 2,3-butanediol (*Saccharomyces cerevisiae*) [24], succinate (*S. cerevisiae*) [25], malonyl-CoA (*E. coli*) [26], acetyl-CoA (*Synechocystis* sp. PCC 6803) [27], ethanol and isobutanol (*Synechocystis* sp. PCC 6803) [28], constraint-based strain optimization methods implemented in software packages such as OptForce [29], OptKnock [30], OptGene [31] and CiED [26] have been used.

Experimental evaluations [12, 13, 17] suggest that not all cyanobacteria may be easily genetically engineered for efficient FFA/biofuel production [13, 14, 32]. Genetic engineering efforts are further affected by the scarcity of available cyanobacterial strains, and the lengthy and costly cultivating and engineering processes. Thus, only few cyanobacterial strains have been evaluated for FFA production, and it is highly likely that other natural strains could be a better chassis [33]. Given the vastness of the bacterial diversity, it would be essential to have a computational method that can rapidly screen all potential strains for FFA production to help narrowing the scope of likely candidates for experimental genetic engineering. The steady accumulation of cyanobacterial genome data (more than 120 genomes are sequenced to date) provides an increasingly rich resource that can be used for this purpose in conjunction with available experimental data.

In this study we provide such an *in silico* screening method FFASC. FFASC estimates and ranks the potential of cyanobacterial strains for FFA production, and hence indirectly biofuel production, based on their predicted proteomes. FFASC has been established based on: 1/a compilation of protein orthologous groups (OGs; see definition below) that impact FFA production; 2/a compilation of relevant assessment criteria; 3/the development of an algorithm that uses the criteria derived from OGs to rank candidate chassis strains based on their estimated potential to produce and excrete FFA. We used FFASC to screen and rank cyanobacterial proteomes for this purpose and indirectly screen their potential as candidates for cyanobacterial biofuel cell factories. The FFASC ranking for the top candidates is supported by their phylogenetic relationship, and by additional indirect *in silico* evidence. Thus, our study suggests that FFASC allows selecting the most promising candidates for experimental validation, whereas the established selection criteria might provide useful insight for efficient metabolic engineering. Moreover, although the methodology developed in our study is focused on FFA production, it can be applied in a similar way to other processes (e.g. production of chemicals, fermentation, nutraceutical and pharmaceutical applications) as well as to other bacteria, fungi or plants.

## Results and Discussion

### Establishing properties that are favorable for cyanobacterial FFA cell factory

**Fig. 1:**
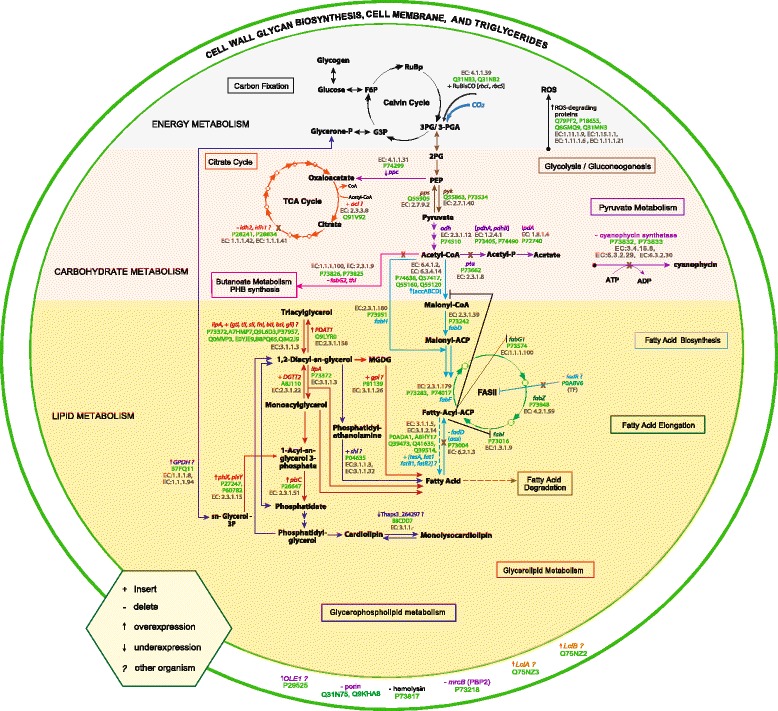
Metabolic map depicting FFA biosynthesis and associated pathways, detailing where 64 proteins impact this process (see Table 1 or Additional file 1: Table S2). Abbreviations: 3-PGA/3PG, 3-phosphoglycerate/3-phosphoglyceric acid; 2PG, 2-phosphoglyceric acid; PEP, phosphoenolpyruvic acid; F6P, fructose 6-phosphate; RuBP, ribulose-1,5-bisphosphate; CO_2,_ carbon dioxide; G3P, glyceraldehyde 3-phosphate; ROS, reactive oxygen species; TCA, tricarboxylic acid; CoA, coenzyme A; ACP, acyl carrier protein; FAS II, type II fatty acid synthases; ATP, Adenosine triphosphate; ADP, adenosine diphosphate

The common procedures used to enhance the biotechnological production of FFA include the introduction of heterologous pathways, as well as the modification of the candidate cell factory metabolism via deletion of genes or enhancing gene expression. However, genetic engineering was not based on the consideration of the collective effects of different criteria that characterize a good cyanobacterial cell factory for FFA production, even though experimental outcomes have shown that not all cyanobacteria are suitable producers [13, 14, 32]. Criteria that would potentially characterize the natural candidate cyanobacterial FFA cell factory include the presence of endogenous FA biosynthesis pathway enzymes [11, 34], as well as associated enzymes that have been modified and tested (through the insertion, overexpression, knockout or knockdown of protein-encoding genes) to increase FFA production in organisms such as algae, cyanobacteria, yeast, *E. coli* and diatoms [11–17, 32, 35–44]. Through a literature search, we identified 64 proteins that are relevant for FFA production. We further classified these 64 proteins into 49 OGs (Table 1, Additional file 1: Table S1), defined here as sets of proteins that are homologous with sufficient domains in common adequate to assume that they affect FA production similarly. To illustrate how these 49 OGs (into which 64 proteins are classified) affect FFA production, in Fig. 1 we show the link of the 49 OGs with the associated metabolic pathways and links to processes associated with energy, carbohydrate and lipid metabolism. Although these 64 proteins cannot be considered complete, they represent the majority of engineering considerations. Based on the results we obtained, it appears these proteins capture many of the relevant characteristics of the organism.

**Table 1:** List of 49 OGs relevant for FFA production

KEGG Orthology	Definition	Effects	Method	Organism	Ref.
rOGs					
K00873 pyk	pyruvate kinase	Carbohydrate metabolism		cyan.	[11, 34]
K01007 pps	pyruvate, water dikinase				
K00161 pdhA	pyruvate dehydrogenase E1 component alpha subunit				
K00162 pdhB	pyruvate dehydrogenase E1 component beta subunit				
K00627 pdhC	pyruvate dehydrogenase E2 component (dihydrolipoamide acetyltransferase)				
K00382 phdD (ipdA)	dihydrolipoamide dehydrogenase				
K00648 fabH	3-oxoacyl-[acyl-carrier-protein] synthase III	Lipid metabolism			
K00645 fabD	[acyl-carrier-protein] S-malonyltransferase				
K09458 fabF	3-oxoacyl-[acyl-carrier-protein] synthase II				
K02372 fabZ	3-hydroxyacyl-[acyl-carrier-protein] dehydratase				
K00208 fabI	enoyl-[acyl-carrier protein] reductase I				
K01046 E3.1.1.3	triacylglycerol lipase	Increase chance of strain to secrete FA	secretion & extraction		[15]
pOGs					
K01962 accA	acetyl-CoA carboxylase carboxyl transferase subunit alpha	Enhance FFA production (Increase supply of desired substrate)	secretion	cyan.	[12, 14, 34, 44]
K01963 accD	acetyl-CoA carboxylase carboxyl transferase subunit beta				
K01961 accC	acetyl-CoA carboxylase, biotin carboxylase subunit				
K02160 accB	acetyl-CoA carboxylase biotin carboxyl carrier protein				
K00432 gpx	glutathione peroxidase	Reduce the toxic effect of FFA production and improve cell growth, physiology and FFA production	secretion	cyan.	[13]
K04564 SOD2	superoxide dismutase, Fe-Mn family				
K06198 coiA	competence protein CoiA				
K03782 katG	catalase-peroxidase				
K03621 plsX	glycerol-3-phosphate acyltransferase PlsX	Lead to higher lipid levels		plant	[11, 35]
K08591 plsY	glycerol-3-phosphate acyltransferase PlsY				
K00655 plsC	1-acyl-sn-glycerol-3-phosphate acyltransferase				
virNOG10454 PDAT1	IQ-domain	Enhancing FA synthesis and diverting FA from membrane lipid to Triacylglycerol	accu.		[36]
virNOG19439 OLEO1	oleosin 1				
K14457 MGAT2	2-acylglycerol O-acyltransferase 2	Enhance acyl-CoA-dependent triacylglycerol TAG			[39]
virNOG24576 LCIA	Anion transporter	Help regulate CO2 intake and increase biomass		algae	[32, 37]
virNOG22763 LCIB	Low-CO2 inducible protein				
K00006 GPD1	glycerol-3-phosphate dehydrogenase (NAD+)	Increase glycerol and neutral lipid content (16- and 18-carbon monounsaturated FA significantly increased)		diatom	[38]
K01601 rbcL	ribulose-bisphosphate carboxylase large chain	Improve FFA production		cyan.	[14, 88]
K01602 rbcS	ribulose-bisphosphate carboxylase small chain				
K01648 ACLY	ATP citrate (pro-S)-lyase	Enhance biofuel precursor production		yeast	[40]
K10804 tesA	acyl-CoA thioesterase I	Remove feedback inhibition and increase production of FFA	secretion	cyan.	[12, 14, 17, 34, 43, 44]
K10781 FATB	fatty acyl-ACP thioesterase B (Plant thioesterase)	Modify the chain length of FFAs for better fuel quality			[11, 12]
K10782 FATA	fatty acyl-ACP thioesterase A	Release FFA			[88]
K14075 PLRP2	pancreatic lipase-related protein 2	Degrade the membrane lipids into FFA with collapse of cell	extraction		[15]
nOGs					
K01595 ppc	phosphoenolpyruvate carboxylase	Increase the lipid content		cyan.	[11]
K01897 aas(fadD)	long-chain acyl-CoA synthetase	Channel needed substrates for synthesis of FFA into divergent or reverse pathways and preventing degradation of desired product	secretion	cyan.	[11–14, 17, 34]
K00059 fabG	3-oxoacyl-[acyl-carrier protein] reductase	Divert energy into production of substantial by-products that would compete with production of FFA			[12]
K00626 E2.3.1.9	acetyl-CoA C-acetyltransferase				
K11003 hlyD	hemolysin D	Enhance secretion of FFA by weakening cell walls			
cyaNOG01264 (PBP2)	penicillin-binding protein	Enhance secretion of FFA by weakening peptidoglycan layer			
K13788 pta	phosphate acetyltransferase	“Channel needed substrates for synthesis of FFA into divergent or reverse pathways and preventing degradation of desired product”			
K13282 cphB	cyanophycinase	“Divert energy into production of substantial by-products that would compete with production of FFA”			
K03802 cphA	cyanophycin synthetase				
cyaNOG01069 porin protein	Carbohydrate-selective porin OprB	Enhanced extracellular FFA concentration			[13]
K13535 CLD1	cardiolipin-specific phospholipase	Increase lipid yields without affecting growth or biomass	accu.	diatom	[41]
K00030 IDH3	isocitrate dehydrogenase (NAD+)	Increase intracellular citrate level which enhance biofuel precursor production		yeast	[40]
K03603 fadR	GntR family transcriptional regulator, negative regulator for fad regulon and positive regulator of fabA	Fatty acid biosynthesis is feadback-inhibited at the transcriptional level by fadR		bacterium	[11, 42]

*Abbreviations*: *rOGs* required OGs, *pOGs*, OGs that positively impact FFA production, *nOGs*, OGs that negatively impact FFA production, *FFA* Free Fatty Acid, *accu.* Accumulation, *cyan*. Cyanobactia

Classification: nOG (based on reported knockout or knockdown) and pOGs (based on reported inserted or overexpressed) during genetic engineering experiments on that organism in order to secretion, extraction, or accumulation fatty acid

In total, we identified 13 OGs (based on reported knockout or knockdown experiments) whose presence in the organisms negatively impacts FFA production. These proteins we collectively named nOG (‘negative OG’; Additional file 1: Table S2). *A*cyl-ACP synthetase/long-chain-fatty-acid CoA ligase (AAS/FadD) is an example of one of the cyanobacterial proteins from this group. Kaczmarzyk and Fulda [45] demonstrated AAS is capable of incorporating exogenous FFA from the culture medium into membrane lipids, an opposite process that reduces FFA production. AAS is also responsible for recovering endogenous FFA released from membrane lipids. *aas* knockout mutants for *Synechocystis* sp. PCC 6803 and *S. elongatus* PCC 7942 (strain SE01) exhibited increased secretion of FFA into the culture medium compared to the wild-type strains [45]. The data suggests that the detected FFA is detached from membrane lipids, and also suggests that AAS plays a role in recycling the released FA, explaining why the presence of the *aas* gene negatively impacts the efficiency of the candidate cell factory.

Based on reported gene insertion and overexpression experiments, we also identified 24 OGs that contain proteins whose presence in the organisms positively impacts FFA production capability (named pOG; Additional file 1: Table S2). Thioesterase (*TesA*) is an example from this group. It was previously demonstrated that *TesA* cleaves the acyl-carrier-protein from the FA moiety, and in this manner increases FA biosynthesis in *E. coli* by reducing feedback inhibition [46]. Thus, Ruffing and Jones [17] cloned the *E. coli-*derived truncated thioesterase (‘*tesA*) and inserted it into the *S. elongatus* PCC 7942 genome along with the *aas* knockout, thereby generating a mutant strain SE02. SE02 produced a higher percentage of saturated FFA and a lower percentage of unsaturated FFA compared to the wild type [17]. Thus, the presence of ‘*tesA* positively impacted the efficiency of the biofuel production. The remaining 12 OGs identified are required for FA production, but are not included in pOG, and we named them rOG (‘required OGs’). The difference between these two groups is that rOGs are essential for FFA production, while pOGs can be considered as ‘enhancers’.

Based on these 49 OGs and their subgrouping to nOG, pOG and rOG, we derived criteria for assessment of suitability of an organism for FFA production (see Materials and Method section, subheading FFASC). In order to estimate an organism’s potential for FFA production, we used all of these derived criteria to generate an overall score that reflects FFA potential. For this purpose we developed FFASC. Our optimization process through which we estimated the optimized weights of the criteria used, is based on two species, *Synechocystis* sp. PCC 6803 and *Arthrospira* (*Spirullina) platensis* NISE-39. Thus, our estimated weights are skewed and not optimal. However, they still provide better qualitative ranking of species for FFA production potential than in the case when all weights are assumed to be equal (see Additional file 1: Table S10). These weights could be improved when more confirmed FFA-producing strains become available for this type of study.

### Screening cyanobacterial proteomes by FFASC

To evaluate the FFA production potential of the 120 cyanobacterial strains that have not been considered for FFA/biofuel production and the five cyanobacterial strains included in the reference dataset, the proteomes of all 125 cyanobacterial strains were screened using FFASC. The number of protein hits obtained from the sequence homology and domain search were used as an input to generate the OG hit numbers associated with each OG, and then applied to the derived set of criteria (weight optimization and ranking algorithm) to predict suitability of cyanobacterial strains for FFA production. The strains were ranked based on the sum of scores generated by all criteria. The higher the score, the better the rank (Table 2).

**Table 2:** Ranked list of cyanobacterial strains based on their FFA production potential score

Ranking position	Ranked species	Values
1	*Prochlorococcus marinus* MIT 9211	1.000000
2	*Prochlorococcus marinus subsp. marinus* CCMP1375	0.999132
3	*Prochlorococcus marinus subsp. pastoris* CCMP1986	0.986870
4	*Prochlorococcus marinus* MIT 9301	0.986697
5	*Prochlorococcus marinus* MIT 9215	0.985005
6	*Candidatus Atelocyanobacterium thalassa* (isolate ALOHA)	0.979893
7	*Prochlorococcus marinus* NATL2A	0.978688
8	*Prochlorococcus marinus* NATL1A	0.978592
9	*Synechococcus* sp. CB0101	0.978368
10	*Synechococcus* sp. RS9917	0.975490
11	*Prochlorococcus marinus* MIT9312	0.974863
12	*Prochlorococcus marinus* MIT 9202	0.973976
13	*Prochlorococcus marinus* MIT 9515	0.973275
14	*Thermosynechococcus elongatus* BP-1	0.968580
15	*Synechococcus* sp. WH 8109	0.966391
16	*Synechococcus* sp. WH 5701	0.965687
17	*Prochlorococcus marinus* AS9601	0.964991
18	*Thermosynechococcus sp.* NK55	0.962108
19	*Synechococcus* sp. JA-3-3Ab	0.957499
20	*Synechococcus* sp*.* CB0205	0.956602
21	*Synechococcus* sp. PCC 7002^**+**^	0.951221
22	*Synechococcus* sp. WH 7805	0.947124
23	*Synechocystis* sp. PCC 6803^**+**^	0.938174
24	*Synechococcus* sp*.* WH 8016	0.933825
25	*Synechococcus* sp. JA-2-3B	0.931812
26	*Cyanobium gracile* PCC 6307	0.931077
27	*Synechococcus* sp. BL107	0.929529
28	*Synechococcus* sp. RS9916	0.929529
29	*Synechococcus* sp. CC9902	0.928199
30	*Synechocystis* sp. PCC 6803 PCC-N	0.922843
31	*Cyanobium* sp. PCC 7001	0.921061
32	*Synechococcus* sp. WH 7803	0.916500
33	*Synechococcus* sp. CC9605	0.916340
34	*Synechococcus* sp. WH 8102	0.887757
35	*Prochlorothrix hollandica* PCC 9006	0.885889
36	*Synechococcus elongatus* PCC 6301	0.883513
37	*Synechococcus elongatus* PCC 7942^**+**^	0.883513
101	*Arthrospira platensis* NIES-39^*^	0.432198
123	*Lyngbya* PCC 8106 (CCY9616)*	0.006115

The list includes all cyanobacterial strain that rank above *S. elongates* PCC 7942 and all reference strains (for the full set see Additional file 1: Table S8). Positive reference strains are marked with superscript + and negative reference strains with *

Even though a limited number of cyanobacterial strains have been engineered as FFA/biofuel producers, several trends can be identified. Wild type *Synechococcus* sp. PCC 7002, *Synechocystis* PCC 6803 and *Synechococcus elongatus* PCC 7942 are reported to produce approximately 2.5 [14], 1.8 [12] and 0.3 [14] mg/L of FFA, respectively. However, these criteria are generally not sufficient to identify the putative chassis strains. Ruffing [14] has demonstrated that *Synechococcus* sp. PCC 7002 is a superior host strain compared to *S. elongatus* PCC 7942 regarding biomass growth rate, environment tolerance, FFA tolerance and production. The ‘*tesA*-expressing *aas*-deficient mutants’ of *Synechococcus* sp. PCC 7002, *Synechocystis* PCC 6803 and *Synechococcus elongatus* PCC 7942, showed an increase in FFA concentration of 40 [14], 83.6 [12] and 29.3 [14] mg/L, respectively, indicating that the increase in FFA concentration depends on the favorable traits in each organisms overall genetic make-up. An additional genetic manipulation, that is, the overexpression of Rubisco, in *Synechococcus* sp. PCC 7002 further increased the FFA concentration to 103 mg/L. To-date the strain with the most genetic manipulations is *Synechocystis* PCC 6803, which yields the highest FFA concentration of 197 mg/L. However, its genetic modifications include weakening of the cell wall layers that may affect survival capabilities under adverse conditions [12]. It was also demonstrated that while engineered *S. elongatus* PCC 7942 strains successfully produce and secrete FFA, these cells are compromised with a decrease in Chl-*a* content and photosynthetic yield, as well as changes in pigment localization that may be partially attributed to the unsaturated FFA being oxidized into toxic products [17]. Such cell physiology associated ramifications are not known for engineered *Synechocystis* sp. PCC 6803. However, engineered *Synechocystis* PCC 6803 were reported to mainly produce saturated FFA. These potential differences in the host metabolism suggest that *Synechocystis* sp. PCC 6803 may be a better chassis strain for FFA production than *S. elongatus* PCC 7942. Nonetheless, both *Synechocystis* PCC 6803 and *S. elongatus* PCC 7942 are fresh water strains. On the other hand, marine strain *Synechococcus* sp. PCC 7002 has been shown to endure salt concentrations up to 1.7M [47], making it an attractive target for large-scale production using marine water based media. *Synechococcus* sp. PCC 7002 may also be the superior chassis strain, compared to both *Synechocystis* sp. PCC 6803 and *S. elongatus* PCC 7942, owing to its short doubling time and remarkable light and temperature tolerance [14]. Additionally, *Lyngbya* sp. PCC 8106 was shown to produce less FFA/biodiesel than *S. elongatus* PCC 7942 [48], while *A. platensis* NIES.39 showed resistance to genetic manipulation [19, 49]. Thus, the positive reference chassis strains include *Synechococcus* sp. PCC 7002 and *Synechocystis* sp. PCC 6803 as they are easily genetically modified and show superior FFA production followed by *S. elongatus* PCC 7942. Thus, *Lyngbya* sp. PCC 8106 and *A. platensis* NIES.39 are considered in this study as negative reference hosts. Due to the limited number of candidate cyanobacterial FFA producers. Moreover, taking into account the reported outcomes for five cyanobacterial species included in our reference dataset, *Synechococcus* sp. PCC 7002 is expected to perform better than both *Synechocystis* sp. PCC 6803 and *S. elongatus* PCC 7942, followed by *Lyngbya* sp. PCC 8106 and *A. platensis* NIES.39.

**Fig. 2:**
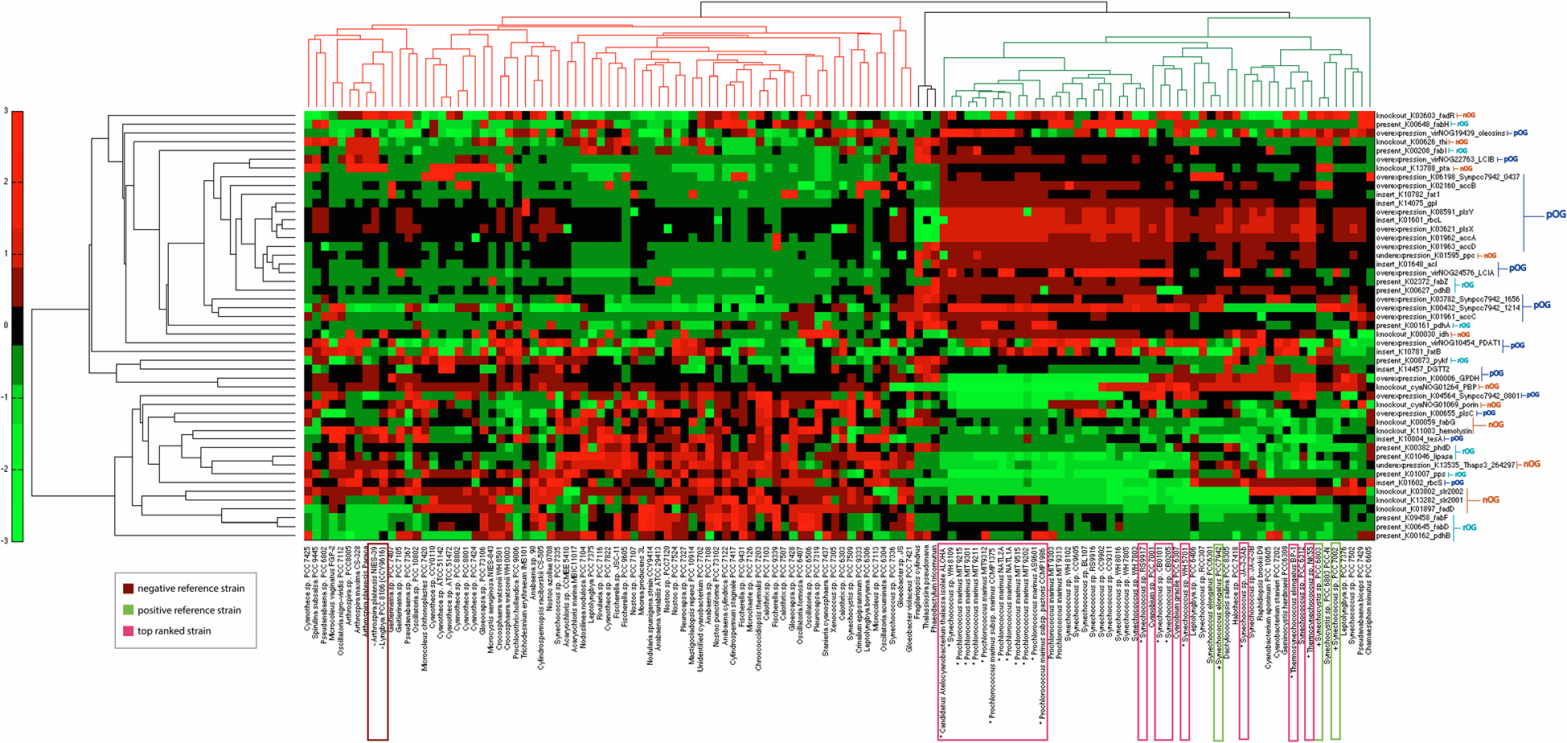
Heatmap visualization of the cyanobacteria screened against the 49 OGs. Clades that contain top ranked strains are represented in green in dendrogram, while the clade that contain the diatoms are represented in black and the clade that contain the negative reference strains are represented in red. Also, positive reference strains names on the x-axis are encircled with green, top ranked strains with maroon and negative reference strains with red

The subsequent list of ranked cyanobacterial strains demonstrates that the positive reference strains rank above the negative reference strains. However, they are not the top ranked strains. The positive reference strains *Synechococcus* sp. PCC 7002, *Synechocystis* sp. PCC 6803 and *S. elongatus* PCC 7942, ranked at position 21, 23 and 37, respectively, while negative reference strains *A. platensis* NIES.39 and *Lyngbya* sp. PCC 8106 ranked at positions 101 and 123, respectively (Table 2). Thus, 36 cyanobacterial strains were ranked above the lowest ranked positive control reference strain at position 37, of which 20 strains (denoted as top ranked strains) ranked above all positive reference strains. All 20 top ranked strains are unicellular. We further observed that the reference strains were ranked as per experimental outcomes reported in the literature. Additionally, weights assigned to criteria after optimization show that 21 of the 49 criteria have the greatest impact on the score and thus the ranking of the strains for FFA production potential (Table 3). However, the criteria impact the score of every strain differently as this impact depends on the composition of the strain’s proteome. We point out that since we are interested in the organism’s natural potential to produce FA, we did not normalize the results for the genome size. We further provide heatmap visualization of the cyanobacteria screened for their potential as FFA producers against the 49 OGs (Fig. 2). The heatmap shows that the majority of the top ranked strains (above *Synechococcus* sp. PCC 7002) are placed in one major clade along with cyanobacterial positive reference strains, while the diatoms, used as an outgroup needed for hierarchical clustering, are placed in a clade of their own. Also, the negative reference strains do not mix with the clade that contain the top ranked strains, that is, the heatmap shows a clear separation between these clades. Moreover, the major clade that contains the top ranked strains generally has a higher number of pOGs (represented by the reddish shaded area) and lower numbers of nOGs (represented by the greenish shaded area), which contrasts with the clade in which negative reference strains are placed. Taken together, the clade with top ranked strains displays more favorable traits for FFA production based on the 49 OGs assessed.

**Table 3:** Weights assigned to rules after optimization that reflect the impact of these rules in the overall scoring

Importance of features	
Features	Weight
overexpression_K00432_Synpcc7942_1214	0.999999981
overexpression_K04564_Synpcc7942_0801	0.999999101
overexpression_K03782_Synpcc7942_1656	0.999998942
overexpression_K02160_accB	0.999998794
present_K00873_pykf	0.999998794
knockout_K11003_hemolysin	0.999998724
underexpression_K13535_Thaps3_264297	0.997856841
knockout_cyaNOG01069_porin	0.946931624
knockout_K01897_fadD	0.921718924
present_K09458_fabF	0.456133041
overexpression_K00006_GPDH	0.396694273
present_K00208_fabI	0.387646822
present_K00161_pdhA	0.314952182
present_K02372_fabZ	0.288150995
overexpression_virNOG24576_LCIA	0.228675187
present_K00648_fabH	0.17462096
present_K00627_odhB	0.168613677
present_K00645_fabD	0.160058392
insert_K01602_rbcS	0.150753918
present_K01046_lipase	0.14966023
overexpression_K06198_Synpcc7942_0437	0.119438174
present_K01007_pps	0.020105541
insert_K14075_gpl	0.013465425
overexpression_K00655_plsC	0.008613511
overexpression_virNOG10454_PDAT1	0.00833575
overexpression_K01963_accD	0.008246186
overexpression_K01961_accC	0.008089475
knockout_K00059_fabG	0.007999865
overexpression_K08591_plsY	0.007682015
overexpression_K01962_accA	0.007630664
insert_K10804_tesA	0.005907112
knockout_K00626_thi	0.004833629
knockout_cyaNOG01264_PBP2	0.004590024
knockout_K03802_slr2002	0.004303632
knockout_K03603_fadR	0.004102976
insert_K01601_rbcL	0.003963175
knockout_K00030_idh	0.003153309
knockout_K13788_pta	0.001763091
overexpression_K03621_plsX	0.001763091
overexpression_virNOG22763_LCIB	0.001763091
present_K00162_pdhB	0.001763091
present_K00382_phdD	0.001763091
underexpression_K01595_ppc	0.001763091
overexpression_virNOG19439_oleosins	0.001299491
knockout_K13282_slr2001	0.00115274
insert_K01648_acl	0.001045169
insert_K14457_DGTT2	0.001001378
insert_K10781_fatB	0.001000657
insert_K10782_fat1	0.001000152

A more in depth assessment of the weights assigned to the 49 OGs (see Table 3) revealed that the medium ranked group (with optimized weights in the range 0.12-0.46) contains mostly the core enzymes of the general fatty acid biosynthesis pathway. These core enzymes are necessary for any producer strain, and their presence cannot be expected to distinguish weak from strong producers. By contrast, the top ranked group (optimized weights in the range 0.92-0.99) contains two principal categories of enzymes: those ensuring precursor availability for biosynthesis of lipids and those involved in handling the oxidative stress associated to FFA synthesis. Belonging to the first category are acetyl-CoA carboxylase [12, 14], pyruvate kinase [11], and acyl-ACP synthetase/long-chain acyl-CoA synthetase [11]. These key enzymes have been validated as metabolic engineering targets for increasing the flux of lipid production [12], and it is not surprising that they have been ranked in the top group. Recently, it was shown that the production of FFAs in cyanobacteria entails the creation of high levels of reactive oxygen species (ROS) which causes oxidative stress, and ultimately loss of membrane integrity [13]. Several enzymes identified in the top group provide relief from oxidative stress and/or are related to membrane permeability: glutathione peroxidase, superoxide dismutase, catalase and porin. Under light, photosynthesis is known to induce the production of ROS which cause lipid peroxidation [50], and the activity of the above-mentioned enzymes can thus also ensure quality control of the produced lipids. A multifunctional lipase was also identified in the top group, coherent with the finding by [51] that stimulating lipid catabolism is required to balance lipid accumulation with efficient growth. The composition of the top group therefore reflects the requirement for the producing cell to handle the flux control points (precursors, lipid accumulation versus biomass accumulation) and to possess enzymes enhancing stress tolerance related to lipid accumulation (ROS/membrane stress tolerance). The weight values obtained during the optimization procedure thus reflect the importance of these two types of key markers for affecting the strain’s potential as cell factories that can be expected to reach a high titer of lipids.

### Comparison between FFASC and Model SEED

**Fig. 3:**
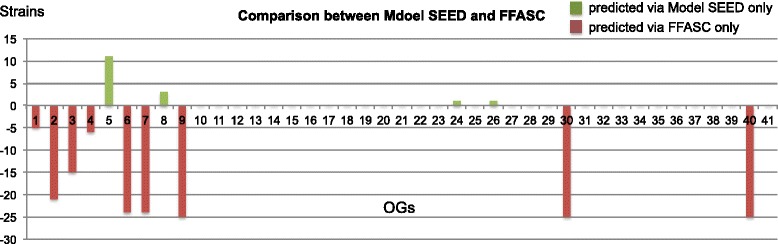
A comparison of the binary (presence/absence) output for the 41 OGs produced by both Model SEED and FFASC. The length of the bar indicates the number of strains with the predicted OG. The absence of bar means the OGs presence/absence for all 25 strains are identical in both methods

Since, Model SEED [52] automatically produces annotations and draft genome-scale metabolic models, we used it here to compare its results with the proposed FFASC approach using the EC numbers corresponding to the 49 OGs that affect FFA production. We found that 41 of the 49 OGs in FFASC can be used for a comparison with Model SEED, as it only focuses on enzymes required for metabolic model reconstruction. Thus, the eight OGs omitted from this analysis include one enzyme that does not have a defined EC number such as EC 3.1.1.-, while other OGs are proteins that do not function as enzymes. For the 41 OGs (Fig. 3), we found Model SEED and FFASC have 28 identical OG hits (68%) for all 25 cyanobacterial strains screened (these are the 20 top-ranked cyanobacterial strains and the five control reference strains). FFASC showed the presence of nine OG hits (22%) that were not present in Model SEED for some species. Similarly, Model SEED showed the presence of four OGs (10%) that were not found to be present using FFASC.

To analyze this data, we tabulated the engineered genes in model organisms *Synechocystis* sp. PCC 6803, *Synechococcus* sp. PCC 7002 and *S. elongatus* PCC 7942, to show the set of genes known to be present in these organisms (see Additional file 1: Table S5). Liu *et al*. [12] made six successive generations of genetic modifications for *Synechocystis* sp. PCC 6803, these modifications include the knockout of *slr2001* and *slr2002*, which encode the cyanophycin synthetases [53]. This shows that *slr2001* and *slr2002* are known to be present in *Synechocystis* sp. PCC 6803, and is reported as present by FFASC, but absent in Model SEED. We further verified that RAST [54] correctly annotated both *slr2001* and *slr2002* in the *Synechocystis* sp. PCC 6803 genome. However, it was omitted from Model SEED, due to the lack of gene-protein-reaction (GPR) association required for incorporation into SEED models. For the four enzymes missing from FFASC, another modification made by Liu *et al*. include the knockout of the *slr1710* (PBP2) gene responsible for peptidoglycan layer assembly [55]. This shows once again that *slr1710* is known to be present in *Synechocystis* sp. PCC 6803, and is correctly found by both Model SEED and FFASC. However, we found that Model SEED identified *slr1710* in 22 additional cyanobacterial strains, whereas FFASC only identified *slr1710* in 11 additional cyanobacteria screened. We found FFASC filtered out the other *slr1710* hits as a consequence of the stringent protein-domain condition applied to increase the accuracy underlying FFASC predictions, that is, only homologous protein sequences that have all domains of the associated protein from the group of 64 proteins were recorded as OG hits. Moreover, all the core enzymes of the general fatty acid biosynthesis pathway were identified using FFASC, whereas Model SEED did not identify FabZ due to the lack of GPR association required for incorporation into SEED models. Here, the differences between Model SEED and FFASC are a consequence of: 1/Model SEED is a generic method in which all pathways are treated equally, whereas FFASC is specialized and focuses on FFA production and is built based on proteins known to either positively or negatively affect FFA production; 2/Model SEED provides the presence or absence of the enzymes, whereas FFASC takes the copy number into account when assessing potential for FFA production; and 3/FFASC include all proteins (not just enzymes) that directly or indirectly affect FFA production. Taken together, FFASC is more refined in assessing the “natural” cyanobacterial strains potential for FFA production, whereas Model SEED was developed for a more generic purpose.

### Additional in silico support for estimated FFA production potential of cyanobacteria

To provide additional support that the predictions obtained by FFASC are reasonable, we used K-means clustering [56] based on the same 49 criteria. To cluster the 128 target species into *k* clusters, where distance of species within a single cluster is minimized and distance between clusters or cluster centers is maximized, a value for *k* has to be set in away that reflects the natural groupings. That is, if *k* is too small, the clustering algorithms will reduce the total number of groups to the specified value of *k*, which forces some natural clusters to combine, thereby producing artificial fusions [57]. Likewise, if the value of *k* is too large, natural clusters will start dividing in an artificial way, to match the specified *k* value.

**Fig. 4:**
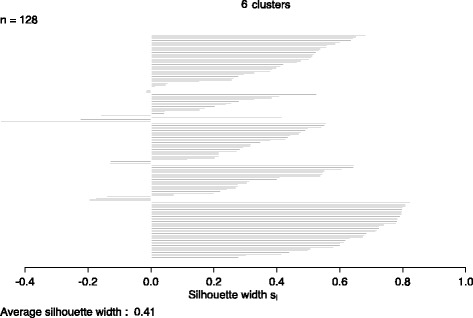
Silhouette plot for clustering quality shows the average silhouette value for clustering 128 species into 6 clusters. A silhouette index ranges from -1 to 1 and a value greater than 0 and closer to 1 indicates that points are in the appropriate cluster

**Fig. 5:**
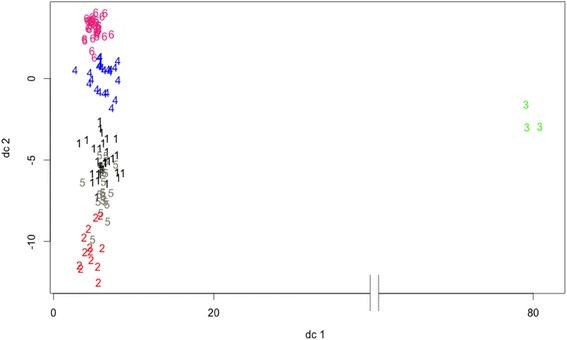
Visualization results of the k-means clustering for the 128 species. The data is projected onto 2D spaces to be able to visualize results using the first two components of the principal component analysis as the axis

To determine the appropriate number of clusters, we take into account that diatoms are eukaryotes and thus act as a type of outlier. When they fall into the same cluster this would indicate the point at which the artificial grouping is omitted [57]. Thus, the clustering will be considered good when diatoms fall into a separate cluster. The number of clusters where diatoms start to group together is *k =* 6 and *k =* 7, the point at which diatoms start to separate is when the number of clusters is *k* = 8. Additionally, using an average silhouette width as the measure of ‘natural’ clustering [57], we found that when considering *k =* 6, 7 or 8, the highest average silhouette width of 0.41 (Fig. 4) was associated with *k =* 6. To further verify the appropriate number of clusters, we also calculated the Calinski-Harabasz (CH) index for *k* = 6 (67.43), *k* = 7 (56.91) and *k* = 8 (61.89) (starting from the point when diatoms cluster together without cyanobacteria, to the point where the diatoms start to separate into different clusters). CH index results verify that *k =* 6 is the appropriate cluster number. A visual illustration of the case *k* = 6 (Fig. 5) shows that cluster 3 is the most distant from the other clusters. This cluster includes the 3 diatoms alone as the outliers, while the negative reference host *Lyngbya* sp. PCC 8106 and *A. platensis* NIES.39 were placed in cluster 5. Top ranked strains, above *Synechococcus* sp. PCC 7002, were all placed in cluster 6. Moreover, all positive reference chassis strains; *Synechococcus* sp. PCC 7002, *Synechocystis* sp. PCC 6803 and *S. elongatus* PCC 7942 were grouped together in cluster 4. Additionally, all strains that ranked below *Synechococcus* sp. PCC 7002 but above *S. elongatus* PCC 7942, were either placed in cluster 6 or 4. The placement of cluster 4 was closest to cluster 6; these clusters slightly overlap one another, but are separate from the other clusters. This indicates that even though K-means clustering does not rank strains, it is still able to discern the potential FFA producers identified with FFASC by clustering them primarily in cluster 6 based on the OG criteria.

Additionally, we note that the three diatoms used in this study are taxonomically distinct (orders *Bacillariales*, *Thalassiosirales* and *Naviculales*), while the 125 cyanobacterial strains are classified under only seven orders, namely *Chroococcales*, *Gloeobacterales*, *Nostocales*, *Oscillatoriales*, *Pleurocapsales*, *Prochlorales* and *Stigonematales* (see Table 4). Only strains of the order *Chroococcales* and *Prochlorales* are found in cluster 6, which seems to contain the best candidates. Strains of the order *Chroococcales* are commonly found in five of the six clusters; however, strains of the order *Prochlorales* were only found in clusters 4 and 6 that include the positive reference strains and top ranked strains. This suggests that *Prochlorales* species may be potentially good FFA producers.

**Table 4:** The analyzed strains classified under their associated order names allocated to the six clusters

Cluster 1	Cluster 2	Cluster 3	Cluster 4	Cluster 5	Cluster 6
Chroococcales	Chroococcales	Bacillariales	Chroococcales	Chroococcales	Chroococcales
Gloeobacterales	Nostocales	Thalassiosirales	Nostocales	Nostocales	Prochlorales
Nostocales	Oscillatoriales	Naviculales	Oscillatoriales	Oscillatoriales	
Oscillatoriales	Pleurocapsales		Prochlorales	Pleurocapsales	
Pleurocapsales	Stigonematales				
Stigonematales					

### Phylogenetic relationships of cyanobacteria

**Fig. 6:**
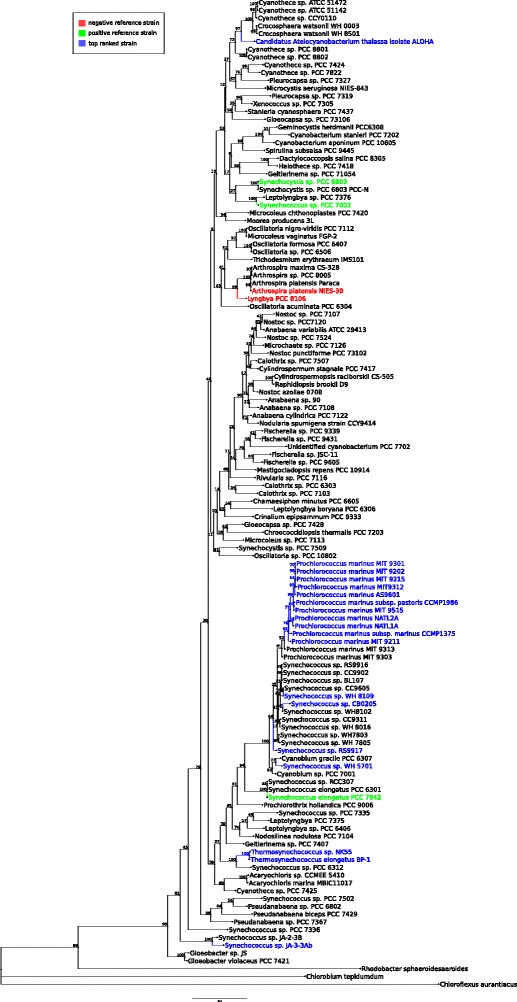
Maximum-likelihood based phylogenetic tree of 124 cyanobacteria and the outgroup using 16S rRNA with bootstrap support. The branches and taxa name for positive reference strains are colored in green and for negative reference strains are colored in red, while the top predicted ranked strains are colored in blue (Table 2)

We explored phylogenetic groupings of 124 cyanobacterial strains used in this study. We found that several of our top ranked candidate cyanobacterial strains are grouped together based on their 16S rRNA. Some exceptions include two *Thermosynechococcus* sp., two *Synechococcus* sp. JA* and *Candidatus Atelocyanobacterium thalassa* (isolate ALOHA) (Fig. 6).

This result is supported by literature, since the top ranked cyanobacterial strains primarily include *Prochlorococcus* (order *Prochlorales*) and marine *Synechococcus* (order *Chroococcales*), which are reported to have diverged from common ancestry [58]. Following the divergence, the *Prochlorococcus* genome is further thought to have ‘streamlined’ [59], thus, the genome size of *Synechococcus* and other cyanobacteria is larger than *Prochlorococcus* genome sizes [60]. Another key feature that differentiates *Prochlorococcus* from *Synechococcus* is their divergent light-harvesting strategies [61]: *Synechococcus* uses the phycobilisome as their light-harvesting antenna that are not found in *Prochlorococcus*. These phycobilisome antenna systems are used by *Synechococcus* to adjust to changes in temperature, likely contributing to its greater geographical occupancy range [62, 63]. Instead, the *Prochlorococcus* main light-harvesting antenna complex is made up of divinyl chlorophyll *a* and *b*, prochlorophyte chlorophyll-binding protein (Pcb), as well as accessory pigment [60, 64]. Collectively, these pigments increase blue light absorption that is the dominant wavelength in deep waters, restricting *Prochlorococcus* to warmer, oligotrophic oceans [65]. Since *Prochlorococcus* is reported to be a leading example of a naturally 'streamlined' genome [59, 66], this suggests that these genomes may require less engineering to efficiently produce high yields of FFA. Moreover, *Prochlorococcus* can be inexpensively cultivated using seawater [67].

Reference strains of the order *Chroococcales,* including *Synechococcus* PCC 7002, *Synechocystis* PCC 6803 and *S. elongatus* PCC 7942, were engineered, and demonstrate the production and secretion of FFA, which provides proof-of-concept. However, none of the predicted top ranked strains of the order *Chroococcales* has been shown to produce FFA. Nonetheless, *Synechococcus* UTEX 2973 (which was not included in this analyses because its genome sequence was not available at the time of this study), has recently been reported to be a fast growing chassis strain for biosynthesis using light and carbon dioxide, growing two times faster than *S. elongatus* PCC 7942 [68]. This finding demonstrates that there are possibly more suitable chassis strains that have not been investigated. Moreover, the Chisholm group [69] have reported that the *Prochlorococcus* strain MIT9313 produces lipid-containing vesicles that are released into the surrounding seawater. These released lipid-containing vesicles maybe collected without disturbing the growth of the *Prochlorococcus*, as opposed to other cyanobacteria or algae that require destroying one batch of cells and starting with a new batch, to retrieve lipids. FFASC ranked the *Prochlorococcus* strain MIT9313 at position 41, suggesting that if the MIT9313 mechanism is a Prochlorococcal trait, there are several other possible vesicle-releasing *Prochlorococcus* strains that may be a better chassis for FFA production. Moreover, the fact that the candidate chassis strains are clustered primarily in orders Synechococcus and Prochlorococcus, is a welcomed surprise that could constitute an additional criterion for positive prediction.

## Conclusion

In this study we developed FFASC, a first screening method that ranks the potential of candidate cyanobacteria for FFA production and excretion based on favorable/non-favorable genetic characteristics. Ranking the candidate species enables narrowing the experimental focus on more likely candidates for good FFA producers. Thus FFASC might prove a useful tool in highlighting candidate strains for industrial-scale biofuel production (based on their natural FFA production potential). The outcome of this analysis suggests unicellular cyanobacterial species such as *Prochlorococcus marinus, Candidatus Atelocyanobacterium thalassa* (isolate ALOHA)*, Synechococcus* sp*.* CB0101, *Synechococcus* sp*.* RS9917, *Thermosynechococcus elongates* BP-1, *Synechococcus* sp*.* WH 8109, *Synechococcus* sp*.* WH 5701, *Thermosynechococcus* sp. NK55, *Synechococcus* sp*.* JA-3-3Ab and *Synechococcus* sp*.* CB0205, as potentially favorable chassis FFA producers. It would also be reasonable to consider other strains with a phylogenetic closeness to the above strains as potential FFA producers as well. Moreover, the methodology developed can be adopted for other metabolic production, and for other species.

We plan to follow-up this research by: 1/expanding the orthologous group to other cyanobacterial genes that are closely related to FFA production such as CO2-fixation, photosynthesis, cell division, environment tolerance genes and 2/develop the FFASC database to classify and evaluate the FFA production potential of cyanobacterial strains based on their proteomes.

## Methods

### Compilation of protein groups that characterize FFA production and excretion

The PubMed database was queried using the query: "biofuel production" OR "free fatty acid production" on 2015/06/30, resulting in 1392 PubMed abstracts retrieved. We conducted a literature search to a compile list of proteins relevant for FFA production from organisms that have been genetically engineered for FFA/biofuel production, as well as proteins required for fatty acid synthesis. In total, we identified 64 such proteins in various organisms including *Escherichia coli*, cyanobacteria, algae, diatoms, plants, and yeast (Additional file 1: Table S1 and S2). These 64 proteins can be classified into 49 OGs, with 43 from KEGG and six from the eggNOG (evolutionary genealogy of genes: Non-supervised Orthologous Groups) [70] database. The 43 KEGG orthology KO identifiers were associated to these proteins using the KOALA (KEGG Orthology And Links Annotation) [71] tool. For the remaining six OGs with no associated KO identifiers, we used eggNOG (Additional file 1: Table S2) to associate OGs to the remaining proteins from the group of 64. All protein sequences included in the 49 OGs were extracted from the UniProt [72] database.

The OGs were categorized as follows (see Additional file 1: Table S2):

- **OGs** **that negatively impact FFA production (nOG):** these OGs contain proteins whose encoding genes have been knocked out or knocked down during genetic engineering experiments to increase the organisms’ potential for FFA production.
- **OGs** **that positively impact FFA production (pOG):** these OGs contain proteins whose encoding genes have been inserted or forced to overexpress to increase the organisms’ potential for FFA production.
- **Required OGs (rOG):** these are a set of proteins required for FA production, not included in pOG.

Based on the effects that the presence or absence of relevant genes have, a set of rules is derived to quantify these effects (see Criteria Generation section).

### Compilation of control and target datasets

#### Control dataset

Our control dataset includes cyanobacteria that have been genetically engineered for FFA/biofuel production. The connection to FA production is that the biodiesel is produced from triacylglycerols that are synthesized from three FAs joined together by one glycerol molecule. However, since there are not many cases of engineered cyanobacteria for FA production, we have also included cyanobacteria *Lyngbya* sp. PCC 8106 in the control set. This strain is not engineered, but it produces biodiesel, although less than *S. elongatus* PCC 7942 [48]. Cyanobacteria that were experimentally shown to be FFA/biofuel producers and have been suggested as candidate biofuel producing cell factories (positive reference strains) include *Synechococcus* sp*.* PCC 7002 [14]*, Synechocystis* PCC 6803 [12, 15, 16], and *S. elongatus* PCC 7942 [17]. On the other hand, those that were experimentally shown not to be promising as FFA/biofuel producers (negative reference strains) include *Lyngbya* sp. PCC 8106 [48] and *A. platensis* NISE-39 [18, 19] (Additional file 1: Table S3). Additionally, diatoms *Phaeodactylum tricornutum* [73, 74], *Thalassiosira psedonana* [41] and *Fragilariopsis cylindrus* [75, 76] were used as outliers required for hierarchical clustering.

#### Target dataset

The target dataset was derived from cyanobacteria. Genome sequences of 125 cyanobacteria were obtained from NCBI [77]. Of these 125 genome sequences collected, 76 are complete genomes and 49 are draft genomes [78] (Additional file 1: Table S4). To standardize the annotation of the 125 cyanobacterial genomes, all genome sequences were re-annotated using the INDIGO pipeline [79] to obtain consistent annotation. Based on that annotation, we derived proteomes of the considered species. The protein sequences were taken in FASTA format.

### Sequence homology and domain search

Protein sequences included in the 49 OGs were mapped to 125 cyanobacterial proteomes using a protein homology search, with the local installation of BLASTp [80, 81], and with an e-value threshold of 0.0001.

We identified 81 conserved protein domain families in the 64 originally identified proteins, using the Pfam database and HMMER[82] with the cut-off gathering threshold (Additional file 1: Table S6). The hidden Markov model (HMM) profiles of these domain families were retrieved from the Pfam database.

**Fig. 7:**
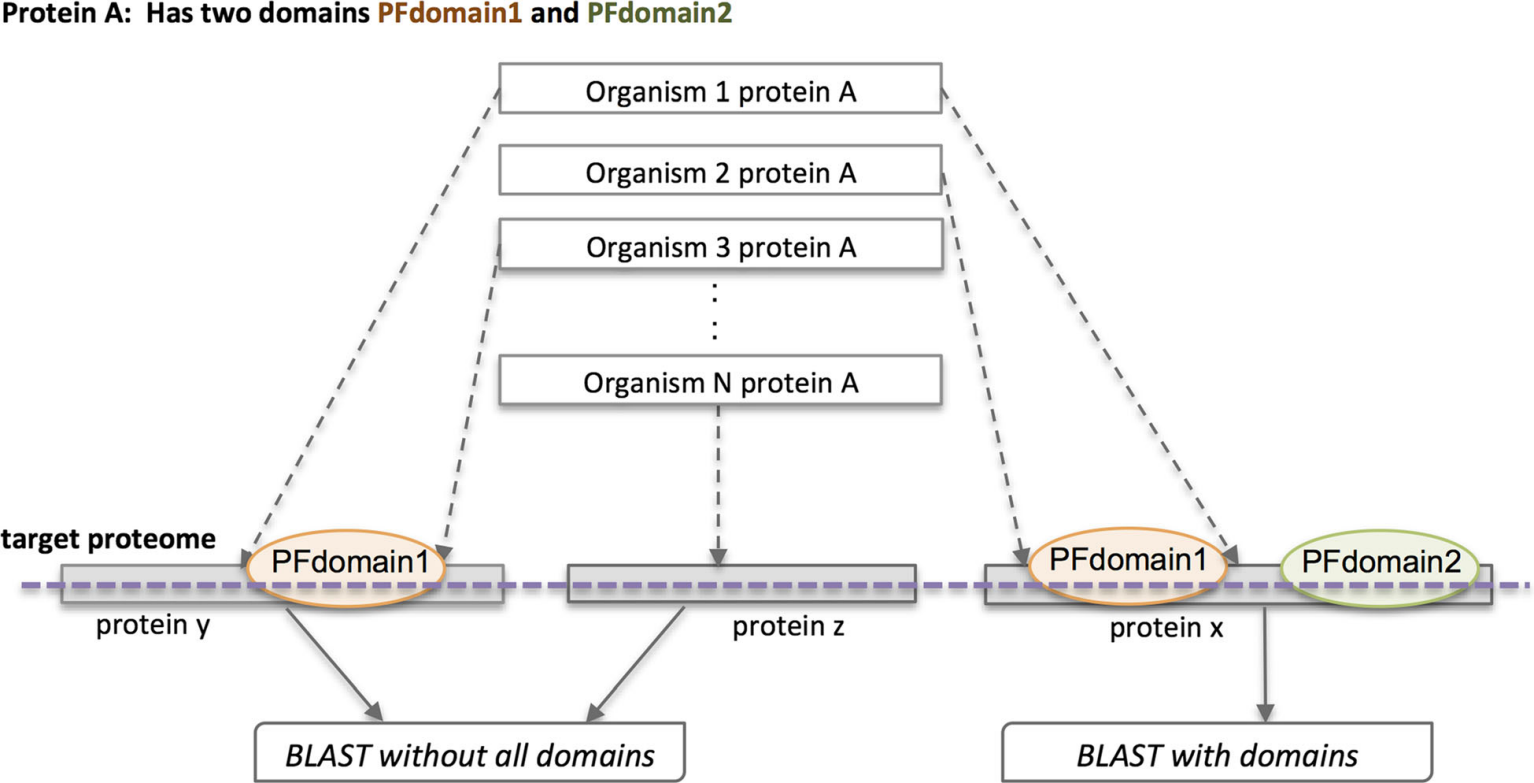
An example to illustrate homologues protein and domains presence and absence. As shown in the figure, if protein A has three homology hits (proteins x, y, and z), the homologous hit of protein A would only be considered if both of its domains (PFdomain1 and PFdomain2) are present in the hit. Hence, only protein x will be used in the analyses (both proteins y and z will be discarded). This stringent rule is applied to filter out weak homology hits obtained by BLAST

The homologous protein sequences identified in the 125 cyanobacterial were further screened with the 81 HMM profiles using a locally installed HMMER [83] program with the trusted cutoff score as a threshold. In the analysis, only homologous protein sequences that have all domains of the associated protein from the group of 64 proteins are used (refer to Fig. 7).

### Criteria generation

In order to provide an integral score of the potential for a species to produce and excrete FFA, we need to quantify the effects of presence or absence of genes that encode for relevant proteins. In our case these will be proteins from different OGs. We consider this quantification as criteria, and we derive one criterion for each OG.

The number of BLASTp hits of all proteins from an OG to the proteome of a species represents an OG hit number (hitN). hitNs are used to define criterion for the OGs. In determining hitNs, only proteins matched by BLASTp that have all domains of the source protein were used. One can conveniently describe species and OGs in terms of hitNs as follows. Suppose that *n* is the number of species and *m* is the number of OGs. We can create an *n* × *m* matrix *C*. In our case *C* is 125 × 49 (see Additional file 1: Table S7). The element (*i,j*) of *C* represents hitN of *j*-th OG in *i*-th species.

**Fig. 8:**
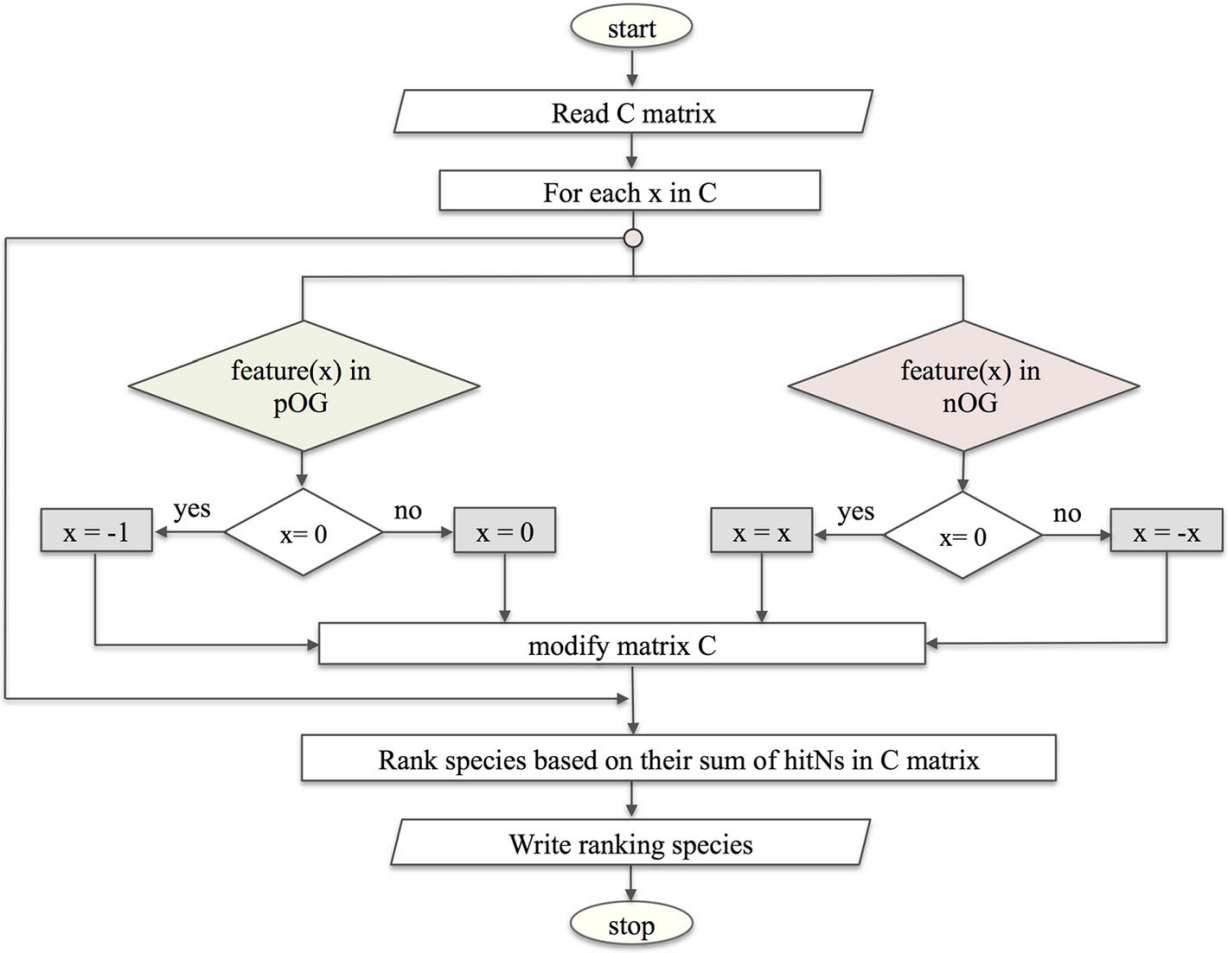
Flowchart of the ranking method employed. It defines the quantification rules based on nOG and pOG (quantification rules for rOG is not illustrated in this method, as rOG receive the values of “hitN”)

The quantification rules are defined as follows. Proteins from nOGs receive the values equal to “–hitN” that correspond to the considered species and the OG. Proteins from pOGs receive the values of “hitN” that correspond to the considered species and the OG. If, however, a pOG has “hitN = 0”, then we assign to it a value of “-1” as a penalty. Proteins from rOGs receive the values of “hitN” that correspond to the species and the OG (Fig. 8).

Consequently, the score that would quantify the potential of species *i* to produce biofuel based on this approach will be described as:

1 $$ score(i)={\displaystyle {\sum}_{j=1}^{49}c\left(i,j\right)}, $$

where *c*(*i,j*) is an element of *C*. While this is not the only possible way to calculate this score we find it simple and suitable. Note that in (1) we assume that all criteria have the same weight equal to 1.

#### FFASC method

1. *Ranking Algorithm (Algorithm 1)*

**Fig. 9:**
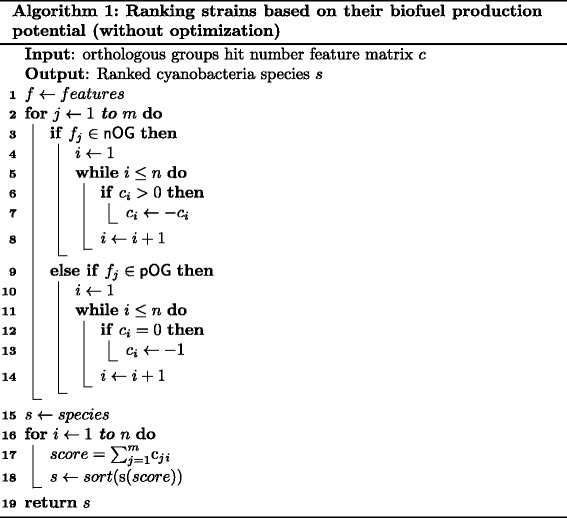
Pseudocode of Algorithm 1

In order to determine scores for each of the species so as to be able to rank them, we will determine the *C* matrix and use it as the input to the algorithm. This algorithm evaluates each of the considered species and generates scores according to (1). Then, the species are ranked, with the higher score being better. The top rank is 1. In this manner we are able to rank the considered species for their FFA production potential based on the scores derived. A pseudo code for the algorithm (Ranking algorithm) is presented in Fig. 9.

2. *Optimization*

In Algorithm 1 we assume that all criteria considered have the same level of influence to the potential of an organism for FFA production as expressed through Equation (1). However, it is reasonable to expect that different criteria have different levels of effects and thus they should have different weights. Because we have no data to determine precisely what values of these weights should be, we used an optimization approach in order to estimate suitable values of these weights. The general ‘constraint’ is that good producers of FA should be ranked higher and well separated from the poor ones. Thus, for the optimization process we selected a positive reference strain, *Synechocystis* sp. PCC 6803 and a negative reference cyanobacteria strain, *A. platensis* NIES.39. The goal of optimization was to make the score difference between these two selected species as big as possible, while having the positive reference strain ranked above the negative reference strain. Optimization was preformed using the pattern search solver (PSS) of the global optimization toolbox in MATLAB. For the PSS, a generalized pattern search algorithm was used with default values. The optimized solutions for the weights found by the optimizer were between 0.001 and 1, where *p* (ranking effect coefficient) is equal to 0.010241 at 1744 iterations, with the objective function value at the solution equal to 0.0232 (convergence level). The proposed objective function to achieve our goal is based on maximizing the difference in scores for the two species used; *Synechocystis* sp. PCC 6803 and *A. platensis* NIES.39 as defined below:

$$ \begin{array}{l}\underset{w}{ \max\ }{w}^T\ast \kern0.5em \left|{x}_1-{x}_2\right|+p \ast \kern0.5em  rank\kern1.75em  where\kern1.25em 1\ge {w}_j\ge 0.001,\ \\ {}\kern16em {\displaystyle \sum }{w}_j=12, \\ {}\kern16em {w}^T\kern0.5em \ast \kern0.5em \left|{x}_1-{x}_2\right|>\kern0.75em 0.001\end{array} $$

Here, *x*_1_ and *x*_2_ are data vectors describing *Synechocystis* sp. PCC 6803 and *A. platensis* NIES.39, respectively, obtained as rows of *C*; *T* denotes the transposition; |()| denotes the absolute value of (); *w* is a weight vector with values indicating the contribution of features as suggested by PSS; *p* is a coefficient to introduce a ranking effect on the optimization; *rank* is the difference in ranking between the *Synechocystis* sp. PCC 6803 and *A. platensis* NIES.39. In this optimization, an optimized set of weights are bounded and constrained as described above. Finally, having optimized the weights, we ranked 125 cyanobacteria, with the scores determined as

2 $$ score\kern0.5em =\kern0.75em {w}^Tx $$

**Fig. 10:**
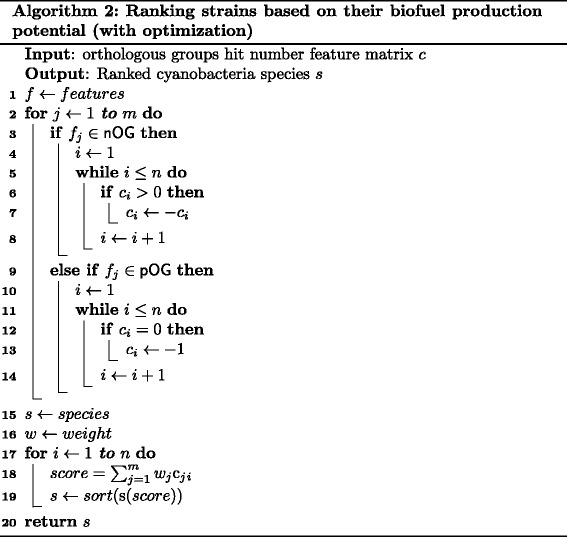
Pseudocode of Algorithm 2

Here, *w* is a column vector of dimension 49. Note that this procedure can be applied to the newly sequenced cyanobacteria species (or other species) added to the set we considered. The pseudocode of Algorithm 2 that describes ranking based on score determined by (2) is presented in Fig. 10.

Based on these optimized weights of different criteria, we propose a list of chassis candidate cyanobacteria strains, where the final ranking reflects the potential of the chassis strain to produce FFA (Additional file 1: Table S8).

### Heatmap generation

We generated heatmap of the produced scores for biofuel production potential for evaluated cyanobacteria and diatoms relative to the 49 OGs. We used the MATLAB 2014a and its function ‘clustergram’ with the following parameters:

- 'Standardize','Row',
- 'Standardize','Column',
- 'Linkage','average',
- 'RowPDist','spearman',
- 'ColumnPDist','spearman';

The matrix *C* was modified following the MATLAB syntax to

*C*+(-0.5 + rand(size(*C*))*10^-10)

by adding a small level of noise to avoid numerical problems with singular matrices.

### Generating data for comparison used in FFASC and Model SEED

The EC numbers corresponding to the 49 OGs were used for comparison with Model SEED. In addition, we submitted 25 cyanobacteria (which include the 20 top-ranked cyanobacterial strains by FFASC and the five control reference strains) to the Model SEED resource (using default values) and obtained the SEED metabolic models and corresponding genome annotations. Similarly, we had binary (presence/absence) output from our FFASC method. We compared the identified EC numbers of 41 OGs in both models and generated the comparison data for Model SEED and FFASC with binary values (0/1) (Additional file 1: Table S5).

We subtracted data for Model SEED from data for FFASC row-wise and obtained values ranging from -25 to 25, where values less than zero indicate the fulfillment of criteria in some strains as required by FFASC only, while values more than zero indicates the fulfillment of criteria in some strains as required by Model SEED only, while zero indicates that the same criteria were required by both FFASC and Model SEED.

### Phylogenetic analyses

In order to see if the ranking obtained as described above reflects any phylogenetic similarities, we performed phylogenetic analyses of cyanobacteria. We used 16S rRNA sequences for the 124 cyanobacterial strains retrieved from INDIGO [79]. *Synechococcus* sp. CB 0101 was not included in this analysis as its 16S rRNA was not available. We also included 16S rRNA of the outgroup (*Chlorobium tepidumdum, Rhodobacter sphaeroides* and *Chloroflexus aurantiacus*). The 16S rRNA sequences for the 124 strains and outgroup were aligned using MAFFT (**M**ultiple **A**lignment using **F**ast **F**ourier **T**ransform) [84] with default parameters on the T-REX Web Server [85]. A maximum likelihood tree [86] was then generated based on the aligned 16S rRNA sequences using **RAxML** (**R**andomized **Ax**elerated **M**aximum **L**ikelihood), with default parameters and 1000 bootstrap runs for the GTRCAT substitution model [87]. The maximum likelihood tree was visualized using FigTree [88] and edited to improve visualization using Inkscape 0.91 [89].

### K-means clustering

To further substantiate the results obtained by applying FFASC, K-means clustering was preformed on the 125 species using all 49 OGs. The K-means procedure in the Package ‘stats’ of R (R 3.1.2) [56] was used. To determine the proper number of *k* clusters, we established 1/the point at which artificial fusions are omitted, that is, when diatoms fall into a separate cluster (determined to be where *k* = 6) and 2/the point at which the natural clusters are divided in an artificial way, that is, when diatoms start to separate into individual clusters (determined as *k* = 8). Thus, based on the properties of the dataset, natural clustering was found to range from cluster 6 to 8 (Additional file 1: Table S9). Further analysis was restricted to natural clusters 6 to 8. To determine the optimal number of *k* clusters from this range, we used the largest average silhouette width as the measure of ‘natural’ clustering and calculating the CH index.

## Additional file

Additional file 1: 
**Table S1.** Classification of orthologous groups. **Table S2.** Proteins used to construct Fig. 1. **Table S3.** Compilation of control dataset. **Table S4.** Constructed target species dataset. **Table S5.** Comparison of FFASC and Model SEED. **Table S6.** Free fatty acid (FFA) protein/enzyme domains. **Table S7.** Orthologous group hit number matrix of 49 OGs and 128 (cyanobacteria and diatom) strains. **Table S8.** Ranked list of 125 strains using FFASC. **Table S9.** 128 strains clustered using K-mean. **Table S10.** Ranked list of 125 cyanobacteria using FFASC without optimization. (XLSX 373 kb)

## Abbreviations

FFA: Free fatty acid
FFASC: Free Fatty Acid SCreen
hitN: OG hit number
nOG: Negative OG
OGs: Orthologous protein groups
pOG: Positive OG
rOG: Required OG

## References

[1] Li J, Liu Y, Cheng JJ, Mos M, Daroch M (2015). Biological potential of microalgae in China for biorefinery-based production of biofuels and high value compounds. N Biotechnol.

[2] Parmar A, Singh NK, Pandey A, Gnansounou E, Madamwar D (2011). Cyanobacteria and microalgae: a positive prospect for biofuels. Bioresour Technol.

[3] Peralta-Yahya PP, Zhang F, del Cardayre SB, Keasling JD (2012). Microbial engineering for the production of advanced biofuels. Nature.

[4] Anemaet IG, Bekker M, Hellingwerf KJ (2010). Algal photosynthesis as the primary driver for a sustainable development in energy, feed, and food production. Mar Biotechnol (NY).

[5] Jones CS, Mayfield SP (2012). Algae biofuels: versatility for the future of bioenergy. Curr Opin Biotechnol.

[6] Cronan JE, Weisberg LJ, Allen RG (1975). Regulation of membrane lipid synthesis in Escherichia coli. Accumulation of free fatty acids of abnormal length during inhibition of phospholipid synthesis. J Biol Chem.

[7] Lennen RM, Kruziki MA, Kumar K, Zinkel RA, Burnum KE, Lipton MS, Hoover SW, Ranatunga DR, Wittkopp TM, Marner WD (2011). Membrane stresses induced by overproduction of free fatty acids in Escherichia coli. Appl Environ Microbiol.

[8] Lennen RM, Pfleger BF (2012). Engineering Escherichia coli to synthesize free fatty acids. Trends Biotechnol.

[9] Liu H, Yu C, Feng D, Cheng T, Meng X, Liu W, Zou H, Xian M (2012). Production of extracellular fatty acid using engineered Escherichia coli. Microb Cell Fact.

[10] Rittmann BE (2008). Opportunities for renewable bioenergy using microorganisms. Biotechnol Bioeng.

[11] Quintana N, Van der Kooy F, Van de Rhee MD, Voshol GP, Verpoorte R (2011). Renewable energy from Cyanobacteria: energy production optimization by metabolic pathway engineering. Appl Microbiol Biotechnol.

[12] Liu X, Sheng J, Curtiss R (2011). Fatty acid production in genetically modified cyanobacteria. Proc Natl Acad Sci U S A.

[13] Ruffing AM (2013). RNA-Seq analysis and targeted mutagenesis for improved free fatty acid production in an engineered cyanobacterium. Biotechnol Biofuels.

[14] Ruffing AM (2014). Improved Free Fatty Acid Production in Cyanobacteria with Synechococcus sp. PCC 7002 as Host. Front Bioeng Biotechnol.

[15] Liu X, Fallon S, Sheng J, Curtiss R (2011). CO2-limitation-inducible Green Recovery of fatty acids from cyanobacterial biomass. Proc Natl Acad Sci U S A.

[16] Liu X, Curtiss R (2012). Thermorecovery of cyanobacterial fatty acids at elevated temperatures. J Biotechnol.

[17] Ruffing AM, Jones HD (2012). Physiological effects of free fatty acid production in genetically engineered Synechococcus elongatus PCC 7942. Biotechnol Bioeng.

[18] NBRC. Arthrospira platensis NIES-39. In: National Institute of Technology and Evaluation (NITE). 2015. http://www.nite.go.jp/en/nbrc/genome/project/annotation/apl.html. Accessed 15 January 2016.

[19] Fujisawa T, Narikawa R, Okamoto S, Ehira S, Yoshimura H, Suzuki I, Masuda T, Mochimaru M, Takaichi S, Awai K (2010). Genomic structure of an economically important cyanobacterium, Arthrospira (Spirulina) platensis NIES-39. DNA Res.

[20] Kaneko T, Nakamura Y, Sasamoto S, Watanabe A, Kohara M, Matsumoto M, Shimpo S, Yamada M, Tabata S (2003). Structural analysis of four large plasmids harboring in a unicellular cyanobacterium, Synechocystis sp. PCC 6803. DNA Res.

[21] Kaneko T, Sato S, Kotani H, Tanaka A, Asamizu E, Nakamura Y, Miyajima N, Hirosawa M, Sugiura M, Sasamoto S (1996). Sequence analysis of the genome of the unicellular cyanobacterium Synechocystis sp. strain PCC6803. II. Sequence determination of the entire genome and assignment of potential protein-coding regions. DNA Res.

[22] Wang B, Wang J, Zhang W, Meldrum DR (2012). Application of synthetic biology in cyanobacteria and algae. Front Microbiol.

[23] Ranganathan S, Tee TW, Chowdhury A, Zomorrodi AR, Yoon JM, Fu Y, Shanks JV, Maranas CD (2012). An integrated computational and experimental study for overproducing fatty acids in Escherichia coli. Metab Eng.

[24] Ng CY, Jung MY, Lee J, Oh MK (2012). Production of 2,3-butanediol in Saccharomyces cerevisiae by in silico aided metabolic engineering. Microb Cell Fact.

[25] Otero JM, Cimini D, Patil KR, Poulsen SG, Olsson L, Nielsen J (2013). Industrial systems biology of Saccharomyces cerevisiae enables novel succinic acid cell factory. PLoS One.

[26] Fowler ZL, Gikandi WW, Koffas MA (2009). Increased malonyl coenzyme A biosynthesis by tuning the Escherichia coli metabolic network and its application to flavanone production. Appl Environ Microbiol.

[27] Anfelt J, Kaczmarzyk D, Shabestary K, Renberg B, Rockberg J, Nielsen J, Uhlén M, Hudson EP (2015). Genetic and nutrient modulation of acetyl-CoA levels in Synechocystis for n-butanol production. Microb Cell Fact.

[28] Erdrich P, Knoop H, Steuer R, Klamt S (2014). Cyanobacterial biofuels: new insights and strain design strategies revealed by computational modeling. Microb Cell Fact.

[29] Ranganathan S, Suthers PF, Maranas CD (2010). OptForce: an optimization procedure for identifying all genetic manipulations leading to targeted overproductions. PLoS Comput Biol.

[30] Burgard AP, Pharkya P, Maranas CD (2003). Optknock: a bilevel programming framework for identifying gene knockout strategies for microbial strain optimization. Biotechnol Bioeng.

[31] Patil KR, Rocha I, Forster J, Nielsen J (2005). Evolutionary programming as a platform for in silico metabolic engineering. BMC Bioinformatics.

[32] Beciri D (2011). Significant biomass increase of genetically altered algae. Bionics news and articles.

[33] Albuquerque NM. Engineering alternative fuel with cyanobacteria. In: Sandia Labs News Releases. 2013. https://share.sandia.gov/news/resources/news_releases/cyanobacteria_fuel/. Accessed 26 July 2015.

[34] Liu T, Khosla C (2010). Genetic engineering of Escherichia coli for biofuel production. Annu Rev Genet.

[35] Janssen HJ, Steinbuchel A (2014). Fatty acid synthesis in Escherichia coli and its applications towards the production of fatty acid based biofuels. Biotechnol Biofuels.

[36] Fan J, Yan C, Zhang X, Xu C (2013). Dual role for phospholipid:diacylglycerol acyltransferase: enhancing fatty acid synthesis and diverting fatty acids from membrane lipids to triacylglycerol in Arabidopsis leaves. Plant Cell.

[37] Miura K, Yamano T, Yoshioka S, Kohinata T, Inoue Y, Taniguchi F, Asamizu E, Nakamura Y, Tabata S, Yamato KT (2004). Expression profiling-based identification of CO2-responsive genes regulated by CCM1 controlling a carbon-concentrating mechanism in Chlamydomonas reinhardtii. Plant Physiol.

[38] Yao Y, Lu Y, Peng KT, Huang T, Niu YF, Xie WH, Yang WD, Liu JS, Li HY (2014). Glycerol and neutral lipid production in the oleaginous marine diatom Phaeodactylum tricornutum promoted by overexpression of glycerol-3-phosphate dehydrogenase. Biotechnol Biofuels.

[39] Sanjaya, Miller R, Durrett TP, Kosma DK, Lydic TA, Muthan B, Koo AJ, Bukhman YV, Reid GE, Howe GA (2013). Altered lipid composition and enhanced nutritional value of Arabidopsis leaves following introduction of an algal diacylglycerol acyltransferase 2. Plant Cell.

[40] Tang X, Feng H, Zhang J, Chen WN (2013). Comparative proteomics analysis of engineered Saccharomyces cerevisiae with enhanced biofuel precursor production. PLoS One.

[41] Trentacoste EM, Shrestha RP, Smith SR, Gle C, Hartmann AC, Hildebrand M, Gerwick WH (2013). Metabolic engineering of lipid catabolism increases microalgal lipid accumulation without compromising growth. Proc Natl Acad Sci U S A.

[42] Lennen RM, Pfleger BF (2013). Modulating membrane composition alters free fatty acid tolerance in Escherichia coli. PLoS One.

[43] Ruffing AM. Metabolic engineering of hydrocarbon biosynthesis for biofuel production. Croatia: INTECH Open Access Publisher; 2013.

[44] Ruffing AM. Liquid, Gaseous and Solid Biofuels - Conversion Techniques(chapter8: Metabolic Engineering of Hydrocarbon Biosynthesis for Biofuel Production). 2013(DOI: 10.5772/52050).

[45] Kaczmarzyk D, Fulda M (2010). Fatty acid activation in cyanobacteria mediated by acyl-acyl carrier protein synthetase enables fatty acid recycling. Plant Physiol.

[46] Cho H, Cronan JE (1995). Defective export of a periplasmic enzyme disrupts regulation of fatty acid synthesis. J Biol Chem.

[47] Batterton JC, Van Baalen C (1971). Growth responses of blue-green algae to sodium chloride concentration. Arch Mikrobiol.

[48] Selvan BK, Revathi M, Piriya PS, Vasan PT, Prabhu DI, Vennison SJ (2013). Biodiesel production from marine cyanobacteria cultured in plate and tubular photobioreactors. Indian J Exp Biol.

[49] Kawata Y, Yano S, Kojima H, Toyomizu M (2004). Transformation of Spirulina platensis strain C1 (Arthrospira sp. PCC9438) with Tn5 transposase-transposon DNA-cation liposome complex. Mar Biotechnol (NY).

[50] Latifi A, Ruiz M, Zhang C-C (2009). Oxidative stress in cyanobacteria. FEMS Microbiol Rev.

[51] Trentacoste EM, Shrestha RP, Smith SR, Glé C, Hartmann AC, Hildebrand M, Gerwick WH (2013). Metabolic engineering of lipid catabolism increases microalgal lipid accumulation without compromising growth. Proc Natl Acad Sci.

[52] Henry CS, DeJongh M, Best AA, Frybarger PM, Linsay B, Stevens RL (2010). High-throughput generation, optimization and analysis of genome-scale metabolic models. Nat Biotechnol.

[53] Ziegler K, Diener A, Herpin C, Richter R, Deutzmann R, Lockau W (1998). Molecular characterization of cyanophycin synthetase, the enzyme catalyzing the biosynthesis of the cyanobacterial reserve material multi‐L‐arginyl‐poly‐L‐aspartate (cyanophycin). Eur J Biochem.

[54] Overbeek R, Olson R, Pusch GD, Olsen GJ, Davis JJ, Disz T, Edwards RA, Gerdes S, Parrello B, Shukla M (2014). The SEED and the Rapid Annotation of microbial genomes using Subsystems Technology (RAST). Nucleic Acids Res.

[55] Marbouty M, Mazouni K, Saguez C, Cassier-Chauvat C, Chauvat F (2009). Characterization of the Synechocystis strain PCC 6803 penicillin-binding proteins and cytokinetic proteins FtsQ and FtsW and their network of interactions with ZipN. J Bacteriol.

[56] Team RC. R: A language and environment for statistical computing. R Foundation for Statistical Computing, Vienna, Austria ISBN 3-900051-07-0. 2013. http://wwwR-projectorg/.

[57] Rousseeuw PJ (1987). Silhouettes: A graphical aid to the interpretation and validation of cluster analysis. J Comput Appl Mathematics.

[58] Dufresne A, Garczarek L, Partensky F (2005). Accelerated evolution associated with genome reduction in a free-living prokaryote. Genome Biol.

[59] Giovannoni SJ, Cameron Thrash J, Temperton B (2014). Implications of streamlining theory for microbial ecology. ISME J.

[60] Scanlan DJ, Ostrowski M, Mazard S, Dufresne A, Garczarek L, Hess WR, Post AF, Hagemann M, Paulsen I, Partensky F (2009). Ecological genomics of marine picocyanobacteria. Microbiol Mol Biol Rev.

[61] Ito H, Tanaka A (2011). Evolution of a divinyl chlorophyll-based photosystem in Prochlorococcus. Proc Natl Acad Sci U S A.

[62] Mackey KR, Paytan A, Caldeira K, Grossman AR, Moran D, McIlvin M, Saito MA (2013). Effect of temperature on photosynthesis and growth in marine Synechococcus spp. Plant Physiol.

[63] Pittera J, Humily F, Thorel M, Grulois D, Garczarek L, Six C (2014). Connecting thermal physiology and latitudinal niche partitioning in marine Synechococcus. ISME J.

[64] Ting CS, Rocap G, King J, Chisholm SW (2002). Cyanobacterial photosynthesis in the oceans: the origins and significance of divergent light-harvesting strategies. Trends Microbiol.

[65] Biller SJ, Berube PM, Lindell D, Chisholm SW (2015). Prochlorococcus: the structure and function of collective diversity. Nat Rev Microbiol.

[66] Sun Z, Blanchard JL (2014). Strong genome-wide selection early in the evolution of Prochlorococcus resulted in a reduced genome through the loss of a large number of small effect genes. PLoS One.

[67] Moore LR, Coe A, Zinser ER, Saito MA, Sullivan MB, Lindell D, Frois-Moniz K, Waterbury J, Chisholm SW (2007). Culturing the marine cyanobacterium Prochlorococcus. Limnol Oceanogr.

[68] Yu J, Liberton M, Cliften PF, Head RD, Jacobs JM, Smith RD, Koppenaal DW, Brand JJ, Pakrasi HB (2015). Synechococcus elongatus UTEX 2973, a fast growing cyanobacterial chassis for biosynthesis using light and CO(2). Sci Rep.

[69] Biller SJ, Schubotz F, Roggensack SE, Thompson AW, Summons RE, Chisholm SW (2014). Bacterial vesicles in marine ecosystems. Science.

[70] Powell S, Forslund K, Szklarczyk D, Trachana K, Roth A, Huerta-Cepas J, Gabaldon T, Rattei T, Creevey C, Kuhn M (2014). eggNOG v4.0: nested orthology inference across 3686 organisms. Nucleic Acids Res.

[71] Kanehisa M, Goto S, Furumichi M, Tanabe M, Hirakawa M (2010). KEGG for representation and analysis of molecular networks involving diseases and drugs. Nucleic Acids Res.

[72] UniProt C (2010). The Universal Protein Resource (UniProt) in 2010. Nucleic Acids Res.

[73] Radakovits R, Eduafo PM, Posewitz MC (2011). Genetic engineering of fatty acid chain length in Phaeodactylum tricornutum. Metab Eng.

[74] Li HY, Lu Y, Zheng JW, Yang WD, Liu JS (2014). Biochemical and genetic engineering of diatoms for polyunsaturated fatty acid biosynthesis. Mar Drugs.

[75] Nordberg H, Cantor M, Dusheyko S, Hua S, Poliakov A, Shabalov I, Smirnova T, Grigoriev IV, Dubchak I (2014). The genome portal of the Department of Energy Joint Genome Institute: 2014 updates. Nucleic Acids Res.

[76] Grigoriev IV, Nordberg H, Shabalov I, Aerts A, Cantor M, Goodstein D, Kuo A, Minovitsky S, Nikitin R, Ohm RA (2012). The genome portal of the Department of Energy Joint Genome Institute. Nucleic Acids Res.

[77] Tatusova T, Ciufo S, Fedorov B, O'Neill K, Tolstoy I (2015). RefSeq microbial genomes database: new representation and annotation strategy. Nucleic Acids Res.

[78] Shih PM, Wu D, Latifi A, Axen SD, Fewer DP, Talla E, Calteau A, Cai F, de Marsac NT, Rippka R (2013). Improving the coverage of the cyanobacterial phylum using diversity-driven genome sequencing. Proc Natl Acad Sci.

[79] Alam I, Antunes A, Kamau AA, Ba Alawi W, Kalkatawi M, Stingl U, Bajic VB (2013). INDIGO - INtegrated data warehouse of microbial genomes with examples from the red sea extremophiles. PLoS One.

[80] Altschul SF, Madden TL, Schaffer AA, Zhang J, Zhang Z, Miller W, Lipman DJ (1997). Gapped BLAST and PSI-BLAST: a new generation of protein database search programs. Nucleic Acids Res.

[81] Mount DW (2007). Using the Basic Local Alignment Search Tool (BLAST). CSH Protoc.

[82] Bateman A, Coin L, Durbin R, Finn RD, Hollich V, Griffiths-Jones S, Khanna A, Marshall M, Moxon S, Sonnhammer EL (2004). The Pfam protein families database. Nucleic Acids Res.

[83] Finn RD, Clements J, Eddy SR (2011). HMMER web server: interactive sequence similarity searching. Nucleic Acids Res.

[84] Katoh K, Kuma K, Toh H, Miyata T (2005). MAFFT version 5: improvement in accuracy of multiple sequence alignment. Nucleic Acids Res.

[85] Boc A, Diallo AB, Makarenkov V (2012). T-REX: a web server for inferring, validating and visualizing phylogenetic trees and networks. Nucleic Acids Res.

[86] Stamatakis A (2014). RAxML version 8: a tool for phylogenetic analysis and post-analysis of large phylogenies. Bioinformatics.

[87] Stamatakis A (2006). RAxML-VI-HPC: maximum likelihood-based phylogenetic analyses with thousands of taxa and mixed models. Bioinformatics.

[88] Ruffing AM (2013). Borrowing genes from Chlamydomonas reinhardtii for free fatty acid production in engineered cyanobacteria. J Appl Phycol.

[89] INKSCAPE Draw Freely. 2004. https://inkscape.org/en/. Accessed 26 Jan 2016.

